# BlasterJS: A novel interactive JavaScript visualisation component for BLAST alignment results

**DOI:** 10.1371/journal.pone.0205286

**Published:** 2018-10-09

**Authors:** Aitor Blanco-Míguez, Florentino Fdez-Riverola, Borja Sánchez, Anália Lourenço

**Affiliations:** 1 ESEI - Department of Computer Science, University of Vigo, Ourense, Spain; 2 CINBIO - Centro de Investigaciones Biomédicas, University of Vigo, Vigo, Spain; 3 Department of Microbiology and Biochemistry of Dairy Products, Instituto de Productos Lácteos de Asturias (IPLA), Consejo Superior de Investigaciones Científicas (CSIC), Villaviciosa, Asturias, Spain; 4 SING Research Group, Galicia Sur Health Research Institute (IIS Galicia Sur), SERGAS-UVIGO, Vigo, Spain; 5 CEB - Centre of Biological Engineering, University of Minho, Braga, Portugal; UMR-S1134, INSERM, Université Paris Diderot, INTS, FRANCE

## Abstract

**Background:**

The wide range of potential applications has made the Basic Local Alignment Search Tool (BLAST) a ubiquitous tool in the field of Molecular Biology. Within this context, it is increasingly appealing to embed BLAST services within larger Web applications.

**Results:**

This work introduces BlasterJS viewer, a new JavaScript library for the lightweight development of Web-based applications supporting the visualisation of BLAST outputs. BlasterJS detaches from similar data viewers by focusing on the visual and interactive display of sequence similarity results and being completely independent of BLAST services. BlasterJS is compatible with the text outputs generated by the BLAST family of programs, namely BLASTp, BLASTn, BLASTx, tBLASTn, and tBLASTx, and works in all major Web browsers. Furthermore, BlasterJS is available through the EBI’s BioJS registry 5, which extends its potential use to a wider scope of bioinformatics applications.

**Conclusions:**

BlasterJS is new Javascript library that enables easy and seamless integration of visual and interactive representations of BLAST outputs in Web-based applications supporting sequence similarity search. BlasterJS is free accessible at http://sing-group.org/blasterjs/.

## Introduction

Sequence similarity searching often provides the first information about newly determined DNA or protein sequences. Specifically, these searches allow the inference of the function of the new sequence from similar (homologous) sequences, i.e. gene or protein sequences that show statistically significant similarity with the new gene or protein (which typically reflects common ancestry).

The Basic Local Alignment Search Tool (BLAST) is one of the most widely used bioinformatics programs [1], and the leading reference in sequence similarity search [2,3]. Current applications include inferring homology from shared sequence similarity, identifying the species associated with an uncharacterised amino acid or DNA sequence, and locating domains shared between proteins.

The public sequence analysis services National Center for Biotechnology Information (NCBI) and the European Bioinformatics Institute (EBI) are widely used, but it is increasingly appealing to embed BLAST services in customised Web applications. Multiple server-side versions of BLAST offer batch query functionality, but visualisation capabilities are poor or too specific. Few Web-based libraries provide customised inclusion of BLAST graphical data viewers in application-specific Web portals and services.

Many times, data viewers are part of BLAST environments, i.e. platforms that offer a number of sequence similarity analysis services. The data viewers provided by EBI and NCBI services fall into this category. Typically, BLAST output viewers are desktop tools, i.e. enable the analysis of BLAST results locally, but do not allow integration in Web applications [4–8]. Other tools are designed as Web servers [9–12], but their integration into other Web applications often requires advanced programming skills. The two exceptions are PLAN [13], which is no longer supported, and Kablammo [10], which offers limited visualisation capabilities. A review on the specifics of existing BLAST data viewers can be found in the S1 Table.

Within this context, this paper introduces BlasterJS, a new JavaScript library for the development of lightweight in-browser data viewers of BLAST outputs. The rationale was to enable the visual display of sequence similarity results while being completely independent of the actual BLAST services. Therefore, the main design principles were (i) compatibility with the text outputs generated by the BLAST family of programs, (ii) minimal integration effort into Web applications, and (iii) visually appealing, customisable and highly interactive data components.

BlasterJS is free accessible at http://sing-group.org/blasterjs/. BlasterJS is also available through the BioJS community-oriented registry to guarantee wide visibility and interoperability with components handling other biological data [14,15].

## Materials and methods

BlasterJS library is entirely written in JavaScript and generates interactive HTML objects that support the display of BLAST alignment results on Web pages. These Web visual components have the advantage of enabling the interactive exploration of data elements, and are thus considered to be user-friendly and provide easily accessible information.

This library is platform independent and works in most commonly used Web browsers, namely Google Chrome, Mozilla Firefox, Opera and Safari.

### Feature set and visual interaction

**Fig 1:**
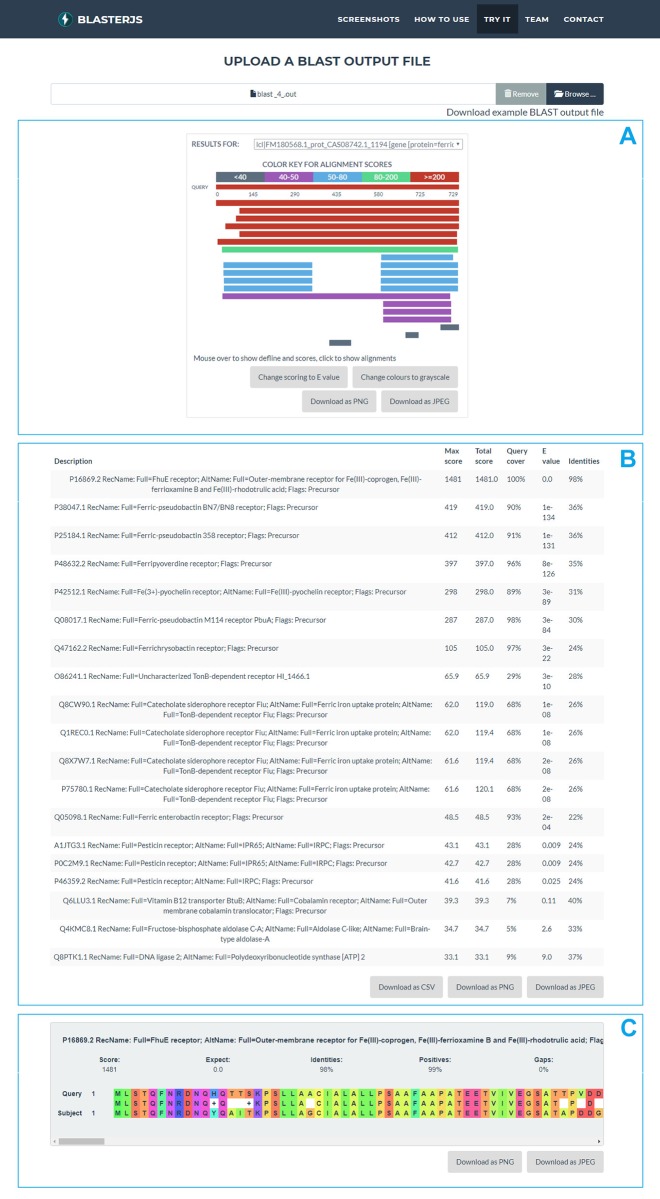
The interactive Web data elements constructed with BlasterJS. Visual components represent (A) the distribution of BLAST hits, (B) the alignment score attributes, and (C) the individual alignment high-score pair hits.

The aim of BlasterJS is to offer a visually appealing, customisable and highly interactive data viewer that is compatible with the outputs generated by BLAST programs and is easy to integrate in Web-based applications supporting sequence similarity search. Therefore, the BlasterJS library enables the tabular representation of BLAST results, i.e. the list of matching sequences and their scores, as well as the interactive and visual navigation of these results, i.e. an intuitive, visual way of looking into the matches. Specifically, the library enables the generation of three complementary and visual interactive elements (Fig 1).

In-browser data display is enabled by intuitive and interactive HTML objects. One of these objects shows the distribution of BLAST hits on the query sequence (Fig 1A), using a representation inspired by the well-known graphical user interface of the NCBI service (http://blast.ncbi.nlm.nih.gov). That is, sequences are represented as horizontal bars and the matching sequence regions are identified using a colour scheme based on the assigned bit score or expected value.

**Fig 2:**
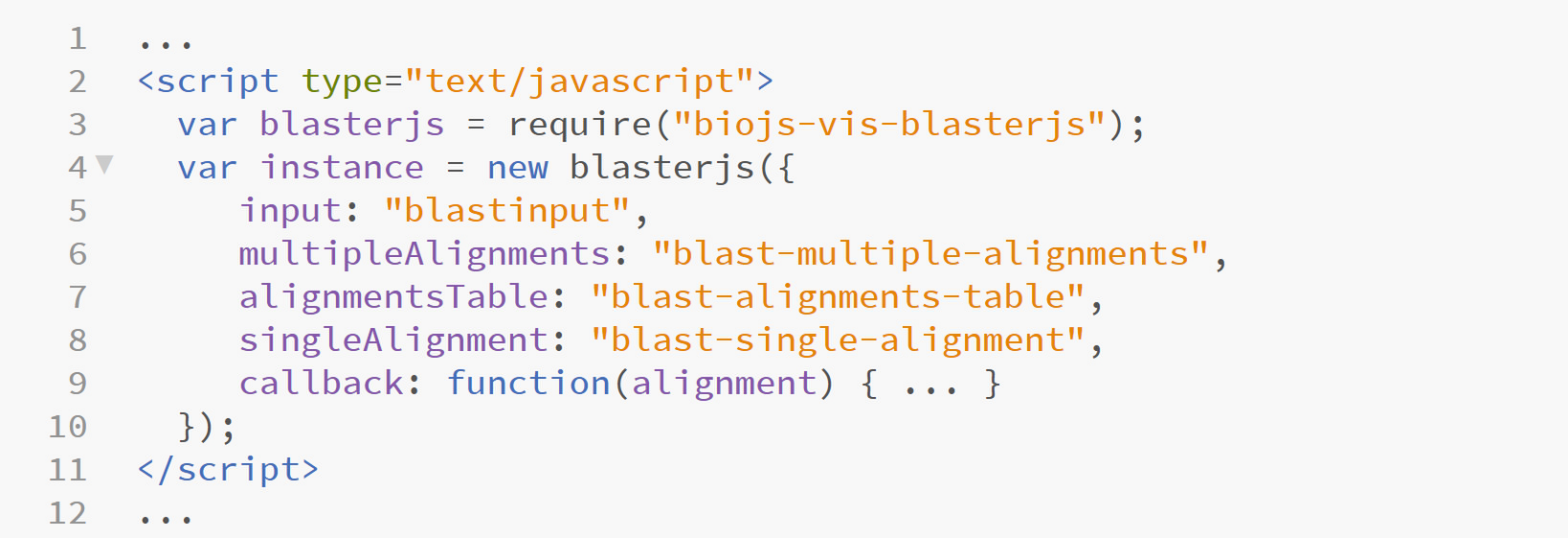
Example of how to set customised callbacks for the alignments table elements in BlasterJS.

Another object provides a tabular description of the high scoring pair attributes, namely the bit score, the expected value, the identity, the positives and the gaps of each hit [11] (Fig 1B). Finally, a third interactive object shows the alignment of each proposed hit with the query sequence at the amino acid/nucleotide level, using the visual presentation of alignments proposed in BioJS MSA (http://msa.biojs.net/) (Fig 1C). As default, the callbacks for the alignment table elements update this third visual element, but BlasterJS also enables the definition of custom callbacks (Fig 2).

### Inputs

BlasterJS is able to parse the text and XML outputs generated by any version of the BLAST+ programs BLASTp, BLASTn, BLASTx, tBLASTn, and tBLASTx [16]. In particular, automated testing is in place to ensure that no errors are introduced into the visualisation while parsing the results table.

**Fig 3:**
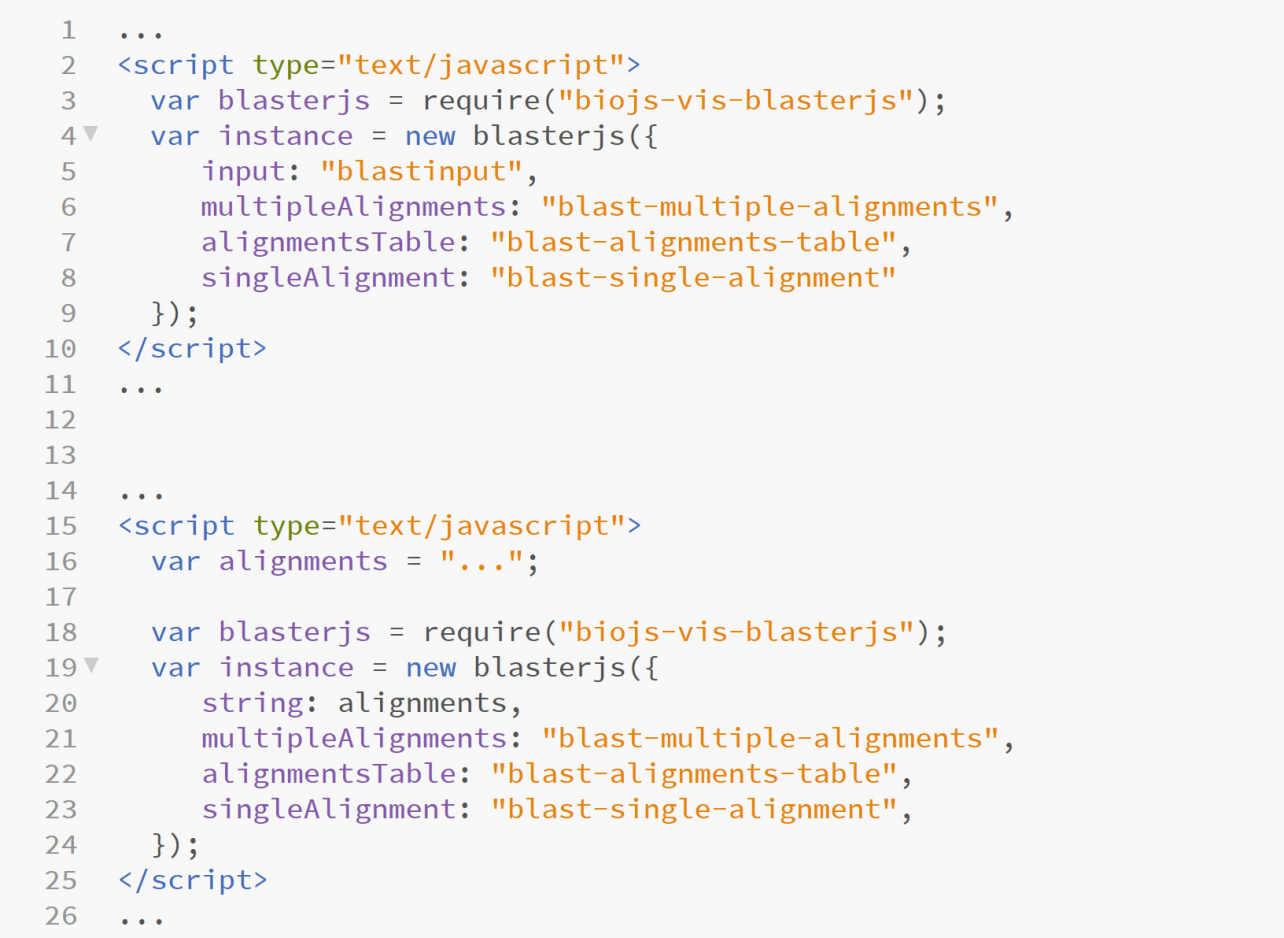
Example of how to upload input data as input element or as string in BlasterJS.

BlasterJS can handle single as well as multiple query BLAST output files. A simple input element, or a string, is used to upload single or multiple query alignment results (Fig 3).

### Application integration and data exportation

**Fig 4:**
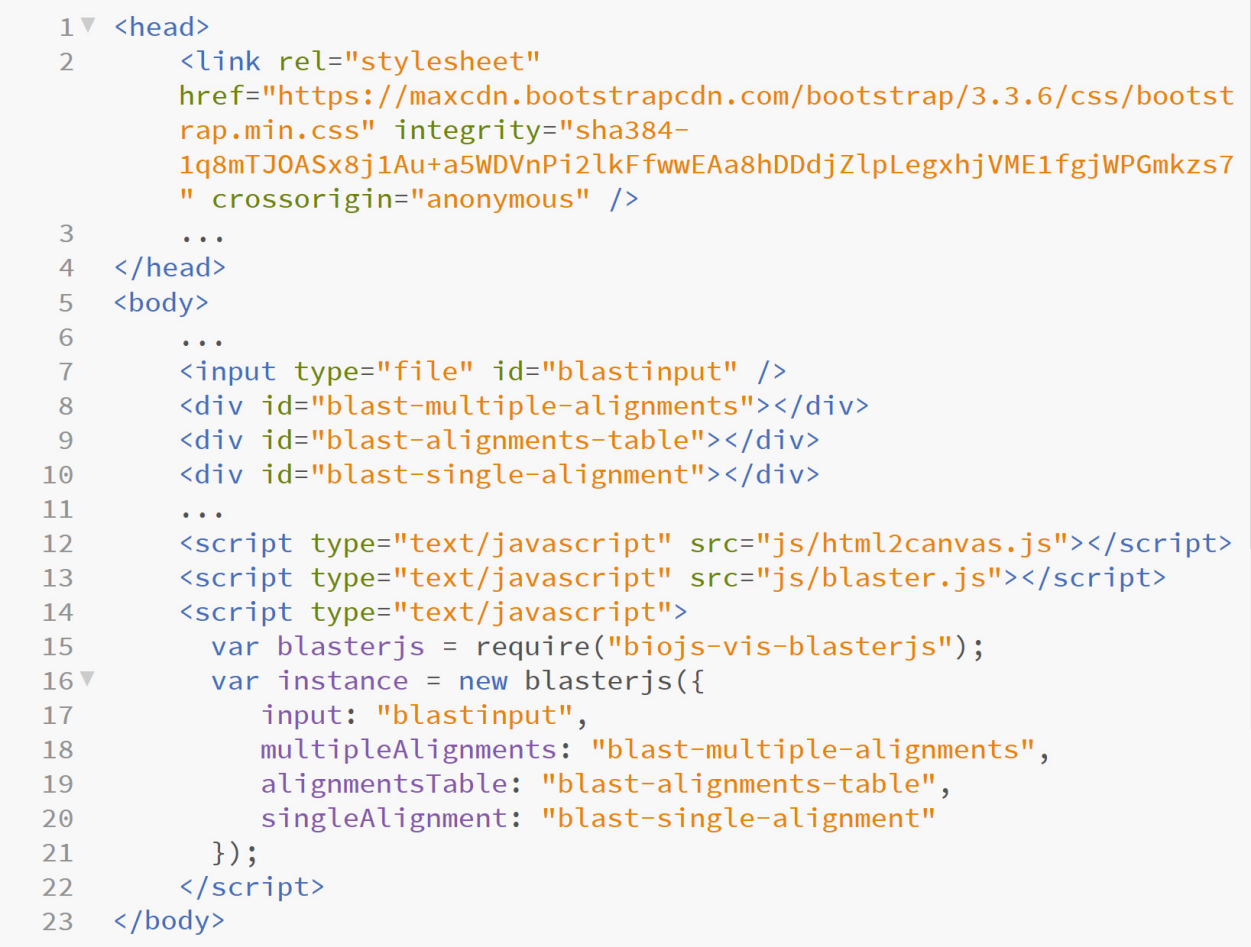
Code snippet of BlasterJS code integration on a Web application. It contains the definition of the visual elements, and the input element responsible for uploading the BLAST output files.

BlasterJS allows programmers with moderate JavaScript programming skills to easily embed visual elements/components in Web application to enable the exploration of BLAST results. The method is quite simple: the programmer has to create an input tag for users to be able to upload BLAST output files and has to specify which visual components the library should produce (Fig 4).

BlasterJS is available through the BioJS registry 5, the centralised repository of BioJS components hosted at the EBI (http://biojs.io/d/biojs-vis-blasterjs). The aim was to grant easy and wide discoverability for the component, while promoting collaborative development with other members of the BioJS community, and reutilisation of BlasterJS by or with any other BioJS components as well as third party applications [15].

In terms of implementation, the general guideline was that code should be compatible with all major Web browsers and CSS compatibility fixes, including any third-party software. The Bootstrap HTML/CSS framework (http://getbootstrap.com/), one of the most-popular front-end component libraries for responsive HTML/CSS/JS developments, ensures the correct visualisation of the Web components. This framework was chosen because of its widespread use as well as for being lightweight and customisable, and having responsive structures and styles. The Html2canvas JavaScript library (https://html2canvas.hertzen.com/) supports the download of HTML objects as JPEG or PNG images, without requiring additional components, such as JQuery components. The alignment data can be downloaded in CSV format.

Comprehensive documentation on how to integrate BlasterJS in application-specific Web services can be found at http://sing-group.org/blasterjs#howto.

## Results and discussion

### Practical integration in MAHMI portal

BlasterJS is currently integrated in the Web portal of MAHMI Database (http://mahmi.org/) [17]. MAHMI database contains a curated collection of empiric data about amino acid sequences with antiproliferative or immunomodulatory bioactivity as well as a data collection produced *in silico* through the systematic processing of publicly available exoproteomes of the human microbiome. Users can use its Web comparative tool, which integrates BlasterJS graphical representation, to inspect the potential immunomodulatory or antiproliferative bioactivity of new amino acidic sequences and identify new, promising peptides. Notably, these peptides are selected based on potential bioactivity against the molecular pathways altered in gastrointestinal disorders of autoimmune and inflammatory nature, such as Inflammatory Bowel Diseases or Colorectal Cancer.

**Fig 5:**
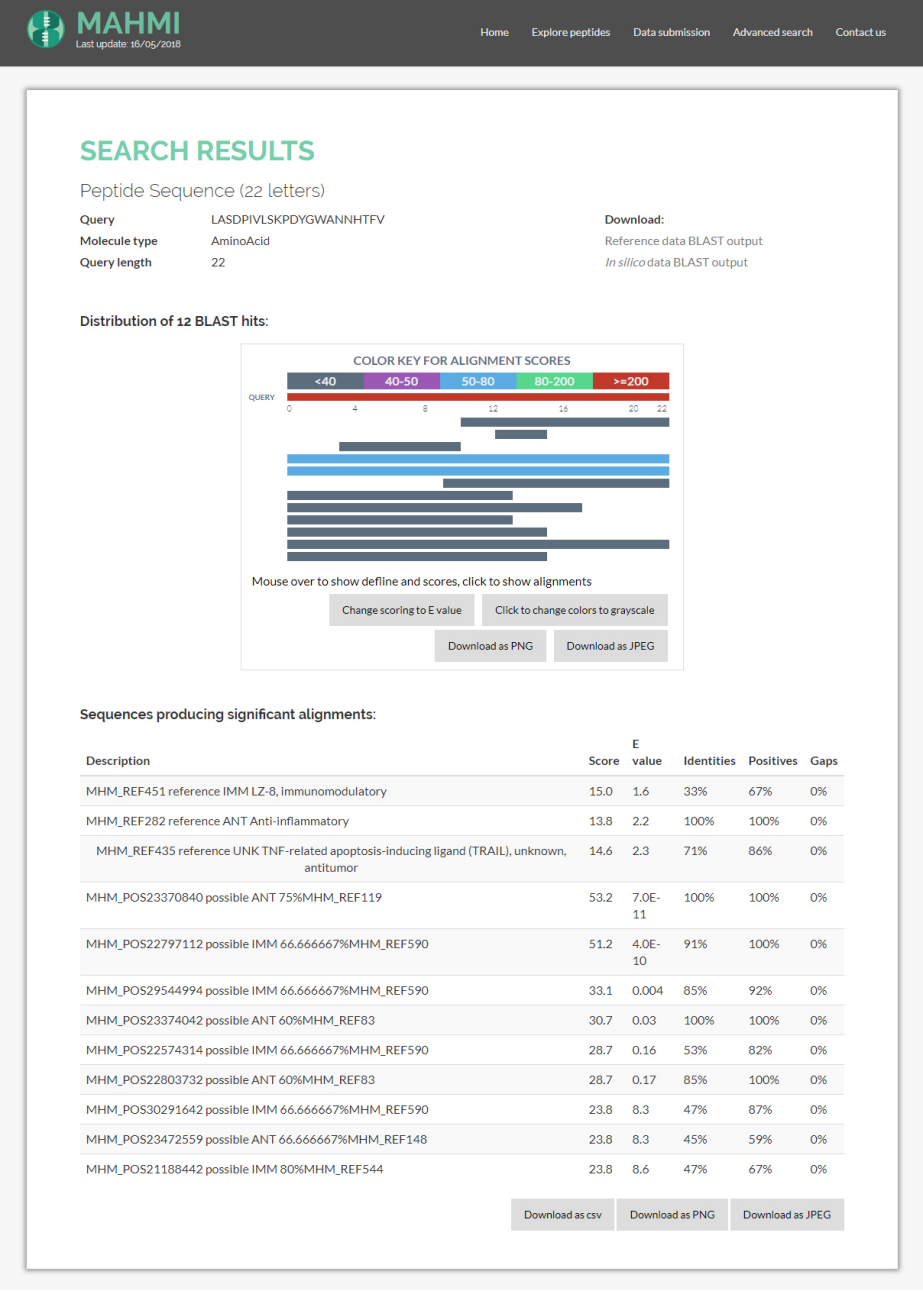
Integration of BlasterJS visual components in MAHMI Web service. This service uses only the two of the data visualisation elements provided by the library, i.e. the distribution of BLAST hits and the alignments table.

As illustrated in Fig 5, BlasterJS graphical components are seamlessly integrated in the Web service of MAHMI. MAHMI Web page embeds the first two elements of BlasterJS to represent any BLAST hits found in the MAHMI peptide database and further describe the alignment of *in silico* and reference peptides in the database against a particular query sequence. Results found for the query peptide sequence LASDPIVLSKPDYGWANNHTFV are shown as example.

### General performance

The performance and memory usage of BlasterJS to render BLAST result files of different sizes was also evaluated. Three BLAST output files, which were produced by the BLASTp tool for three protein datasets of the *Escherichia coli* strain CF073 against the Swiss-Prot database [18], were used: a small dataset of 10 sequences, a medium dataset containing 100 sequences, and a larger dataset having 1000 sequences. The tests considered the rendering of the files by BlasterJS in three main operating systems, i.e. Windows 10, Ubuntu 17.10 and Mac OS X, as well as four different Web browsers, i.e. Google Chrome 67.0, Mozilla Firefox 48.0, Opera 54.0 and Safari 11.0. For each environment, performance was measured in terms of the total execution time and reported along with the JavaScript Heap usage (Table 1).

**Table 1:** Performance analysis of the BlasterJS library. Execution time and JavaScript Heap usage of a small, medium and large BLAST output files were measured over three operating systems using four different Web browsers.

		Ubuntu 17.10			Windows 10			Mac OS X			
		Google Chrome	Mozilla Firefox	Opera	Google Chrome	Mozilla Firefox	Opera	Google Chrome	Mozilla Firefox	Opera	Safari
**10 queries**	**Time**	85 ms	26 ms	116 ms	235 ms	81 ms	260 ms	195 ms	216 ms	200 ms	230 ms
	**JS Heap**	4,5 Mb	4,5 Mb	4,5 Mb	4,4 Mb	4,6 Mb	4,9 Mb	4,8 Mb	4,7 Mb	4,7 Mb	4,7 Mb
**100 queries**	**Time**	530 ms	301 ms	589 ms	1313 ms	802 ms	955 ms	841 ms	627 ms	1026 ms	3540 ms
	**JS Heap**	74,1 Mb	73,5 Mb	73 Mb	70,2 Mb	71,3 Mb	66,8 Mb	64,4 Mb	66,7 Mb	67,3 Mb	66,1 Mb
**1000 queries**	**Time**	3650 ms	2300 ms	4735 ms	8347 ms	4616 ms	5459 ms	6386 ms	114260 ms	6675 ms	12860 ms
	**JS Heap**	405 Mb	401 Mb	396,1 Mb	452 Mb	436 Mb	445 Mb	391,1 Mb	388,3 Mb	382,1 Mb	393,7 Mb

Web browser versions: Google Chrome 67.0, Mozilla Firefox 48.0, Opera 54.0 and Safari 11.0. Machine characteristics: Windows 10, 2.4 GHz Intel Core i7 (4 cores) and 8 Gb RAM; Ubuntu 17.10, 3.6 GHz Intel Core i7 (4 cores) and 16 Gb RAM; and Mac OS X, 2.3 GHz Intel Core i5 (2 cores), 2 Gb RAM.

Regarding operating systems, BlasterJS achieved the best execution times in Ubuntu 17.10. The lower performance of BlasterJS in Mac OS X can be partially explained by the fact that the available machine is quite inferior in resources compared to the other two. Regarding the Web browsers, BlasterJS performs the best in Mozilla Firefox. The execution times in Google Chrome and Opera are quite similar whereas Safari got the worst results (this browser can only be tested over Mac OS X).

Looking into the JavaScript Heap usage the scenario changes. That is, BlasterJS shows the best memory usage results for Mac OS X, especially with the larger file, followed by the results in Windows 10 second, and then, the execution in Ubuntu 17.10. For JavaScript Heap usage, all browsers make a very similar use of resources, with very small differences between them.

## Conclusions

BlasterJS is a new Javascript library devoted to the display of BLAST results. A number of data viewers were already available, but none enabled easy and seamless integration of visual and interactive representations of BLAST results in Web applications. BlasterJS was specifically designed to fulfil this purpose and thus, it is independent of the BLAST service generating the results and requires minimal programming effort. BlasterJS is compatible with the text outputs generated by the BLAST family of programs and provides intuitive, easy-to-navigate graphic data representations, which are compatible with the most commonly used Web browsers, namely Google Chrome, Mozilla Firefox, Opera and Safari.

BlasterJS is freely available under a GNU GPL 3.0 license. The source code and documentation are public at http://sing-group.org/blasterjs/ and https://github.com/sing-group/biojs-vis-blasterjs.

## Supporting information

S1 Table Comparison of BlasterJS to other BLAST data viewers. Notes for selected categories: Interface: General impression of the interface style. Interactive GUI = GUI as in contemporary user-friendly applications such as MS Word. Tabular = output is presented mostly as tables, possible with some limited interaction (sorting by table columns). Integrated BLAST: whether the program supports BLAST searches started from within the program. Multi-query analysis: whether multiple queries can be visualized and analysed in an integrated way. Graphical alignments: how alignments between queries and hits are graphically displayed. Query-hits = one selected query is displayed with the associated hits. Hit-Queries = one selected hit is displayed with its associated queries. High-throughput: whether the program can handle high-throughput BLAST data, i.e. BLAST results with more than 10 000 queries. Splitting and merging files: whether it is possible to split files (and possible merge them subsequently) to handle large data set. Additional features: Program features extending the information originally present in the BLAST output file. BLAST+ compatible: The latest NCBI BLAST implementation (denominated BLAST+) includes a changed BLAST output format. N/A = Not Applicable.
(XLSX)
